# Determinants of Under-Five Pneumonia at Gondar University Hospital, Northwest Ethiopia: An Unmatched Case-Control Study

**DOI:** 10.1155/2019/9790216

**Published:** 2019-09-23

**Authors:** Yordanos Markos, Abel Fekadu Dadi, Abayneh Girma Demisse, Yohannes Ayanaw Habitu, Behailu Tariku Derseh, Getu Debalkie

**Affiliations:** ^1^University of Gondar Specialized Hospital, Gondar, Ethiopia; ^2^University of Gondar, College of Medicine and Health Science, Department of Epidemiology and Biostatistics, Gondar, Ethiopia; ^3^University of Gondar Specialized Hospital, Department of Internal Medicine, Gondar, Ethiopia; ^4^University of Gondar, College of Medicine and Health Science, Department of Reproductive Health, Gondar, Ethiopia; ^5^Debre Berhan University, College of Health Sciences, Department of Public Health, Debre Berhan, Ethiopia; ^6^University of Gondar, College of Medicine and Health Science, Department of Behavioral Health, Gondar, Ethiopia

## Abstract

**Background:**

Pneumonia causes about two million under-five deaths each year, accounting for nearly one in five child deaths globally. Knowing the determinants of under-five pneumonia is useful for prevention and intervention programs that are aimed to control the disease. Thus, the main aim of this study was to assess the determinants of under-five pneumonia at Gondar University Hospital, Ethiopia.

**Methods:**

An institution-based unmatched case-control study was carried out from April 1 to April 30, 2015, taking a sample size of 435 study participants (145 cases and 290 controls). The researchers used a systematic random sampling technique for selecting cases and controls. Data were entered and cleaned using Epi Info version 7 and exported to SPSS version 20 for analysis. Bivariable analysis was performed, and variables with a *p* value less than 0.2 were entered into multivariable logistic regression. Determinant factors were identified based on *p* value less than 0.05 and adjusted odds ratio with 95% confidence interval (AOR with 95% CI).

**Results:**

An increased odds of pneumonia was associated with children who had diarrhea in the past fifteen days of data collection (AOR = 6.183; 95% CI: 3.482, 10.977), children's mothers who did not hear about how to handle domestic smoking (AOR = 5.814; 95% CI: 2.757, 12.261), and children of mothers who did not follow proper handwashing practice (AOR = 3.469; 95% CI: 1.753, 6.863).

**Conclusions:**

Being infected with diarrhea, not knowing how to handle domestic smoking, and poor compliance with proper handwashing practice were identified as determinants of pneumonia. Dedicated, coordinated, and integrated intervention needs to be taken to enhance proper handwashing practice by mothers/caregivers, improve the indoor air quality, and prevent diarrheal diseases at the community level.

## 1. Introduction

Pneumonia is an acute illness of the lung alveolar air spaces. It can be caused by many pathogens, but the majority of severe cases are caused by bacteria, of which the most important agents are *Streptococcus pneumoniae* and *Haemophilus influenzae* [1].

Globally, 1.9 million under-five children die from pneumonia every year [2], and it is the leading infectious cause of death among under-five children, killing an estimated 935,000 children each year. It causes 15% of all deaths among under-five children; 2% of which are newborns [3–6].

The 2013 annual report of UNICEF showed that half of all under-5 deaths occur in only five countries: India, Nigeria, the Democratic Republic of the Congo, Pakistan, and China. Most deaths were due to infectious and preventable diseases. Pneumonia, diarrhea, and malaria together killed roughly 2.2 million under-five children in 2012, accounting for a third of all under-five deaths [7].

The fourth MDG call was to reduce child mortality by two-thirds between 1990 and 2015, nevertheless the morbidity and mortality of pneumonia still remain a public health problem in developing countries. Sub-Saharan Africa had the highest under-five mortality rate, having an average under-five mortality rate of 172 deaths per 1,000 live births. Consistently, pneumonia is a major cause of morbidity and mortality among under-five children in sub-Saharan Africa [8, 9].

In Ethiopia, pneumonia, diarrhea, and malaria are the three major causes of death among under-five children. Pneumonia is one of the leading causes of mortality among under-five children in the country, contributing 28 percent of death [10].

Emerging evidence has shown that children are at greater risk of dying before age five if they are born in rural areas, poor households, or to a mother denied basic education. Though under-five mortality was consistently reduced over the past 20 years, few progresses in reducing neonatal mortality have been done [11].

Even though some studies conducted previously found out factors like housing [12, 13], weight [14], residence [15–17], maternal age [15–17], parental educational status [12, 18, 19], economic status of parents [15–17], sex of child [15–17], child age [16, 20], nutritional status of child [21–24], history of past morbidity [16, 25, 26], history of vaccination [27, 28], birth, and environmental factors [29] as factors associated with under-five pneumonia, they did not clearly illustrate the association. We used a different study design that is unmatched case-control study design to fill the gap that other studies did not employ. The findings of this study might help as an entry point for health-care providers, planners, and stakeholders who work to diminish under-five pneumonia and its related complications.

## 2. Methods

### 2.1. Study Setting and Design

The institution-based unmatched case-control study design was carried out from April 1 to 30, 2015. The study was conducted in Gondar University Hospital (GUH), which is a referral and teaching hospital, serving for around 5 million people. It is one of the government hospitals in Amhara Regional State. GUH has 518 total numbers of beds. The source population was all under-five children who visited Gondar University Hospital pediatrics OPD, and the study population was all under-five children who visited Gondar University Hospital pediatrics OPD during the data collection period.

  Cases: all under-five children with diagnosed pneumonia who came for treatment service during the data collection period.
  Pneumonia: all under-five cases visited the hospital with cough or difficulty of breathing and age-specific fast breathing or consolidation or infiltration that are found positive on chest X-ray.
  Controls: all under-five children who have sought care other than pneumonia who visited GUH under-five OPD during a similar study period.

### 2.2. Sample Size Determination and Sampling Procedure

The sample size was calculated using Epi Info version 7 for unmatched case-control study design based on the assumptions that the child age between 24 and 34 months is a significant predictor of under-five pneumonia. From a previous case-control study, 28% of controls and 42.7% of cases were children whose ages were between 24 and 34 months. The level of significance was 0.05, the power of the test was (1−*β*) = 80%, and the control to case ratio was 2. The proportion of exposure among controls (p1) = 28% with the proportion of exposure among cases (p2) = 42.7% and AOR = 1.92 was inserted to the Epi Info formula to determine the sample size. Accordingly, after considering 10% nonresponse, the final sample size was 145 cases and 290 controls [16]. The estimated number of under-five children outpatient department visit for six months was 8,417. Among these, the number of under-five pneumonia cases was 843; taking the average number of pneumonia case per one month (843/6 = 141), all cases were taken for the study as a case. This means all under-five pneumonia cases who visited U5-OPD during the data collection period (April 1 to 30, 2015) were included in the study until the sample size was reached. However, systematic random sampling was used to select controls for this study. The estimated number of controls for the past six months prior to data collection was 7574 (8417−843 = 7574). Dividing the controls to six months, we got the average number of controls for one month (7574/6 = 1262). Therefore, the sampling fraction, *k* = 1262/290 = 4, meaning every 4^th^ member of the control was selected. Finally, a random number from 1 to 4 was selected to recruit the first control.

### 2.3. Data Collection Tool and Data Quality Control

Parents of children aged two months to five years who were on-site during data collection were eligible to participate in the study. Researchers used a pretested, structured Amharic questionnaire to collect information. Face-to-face interviews and document review was done to collect data on sociodemographic and independent variables. Anthropometric measurement of height (to the nearest 0.1 centimeters) and weight (to the nearest 0.1 gram) of the children were taken. Nutritional status of the children was determined using Enhanced Nutritional Action (ENA) software. The physician examined the patients as cases and controls based on chest X-ray result. The interview was carried out in a private room, which was prepared near the child OPD.

### 2.4. Data Processing and Analysis

All data were checked, coded, entered to Epi Info 7, and analyzed using SPSS version 20. Researchers checked the extent of outliers, the different statistical assumptions, and the appropriate correction mechanisms prior to analysis. Association of each independent variable was assessed with binary logistic regression, and the strength of their association was computed by an unadjusted odds ratio (COR, 95% CI). Variables showing statistically significant associations with the outcome variables at (*p* ≤ 0.2) were considered as potential risk factors for pneumonia [30] and simultaneously subjected to stepwise multiple logistic regression models to determine independent risk factors of pneumonia. A *p* value < 0.05 was considered statistically significant. Multicollinearity test was done to check whether there were correlated independent variables.

## 3. Results

### 3.1. Sociodemographic Characteristics

All consented mothers have participated in the study. The mean age of mothers was 28 years (SD ± 5) for cases and 29 years (SD ± 5) for controls. The mean age of the children was 20.44 months (SD ± 15) for cases and 15.53 months (SD ± 11.8) for controls. Concerning the respondent's religion, 83.4% of controls and 84.5% of cases were orthodox Christian followers and the rest were Muslims and Protestants. Regarding the educational status of mothers, 33.8% of cases and 20.3% controls were illiterate, and 29% of cases and 22.1% of controls' fathers were illiterate (Table 1).

### 3.2. Child and Parental Characteristics

Of all under-five children included in the study, 51% of cases and 47.9% of controls were males. Concerning the nutritional status of under-five children, 82.8% of cases and 40.4% of controls were stunted. Majority of cases (86.9%) and controls (97.6%) were exclusively breastfed for six months. Concerning complementary feeding, 47.6 percent of cases and 78.3 percent of controls started at six months. Sixty-eight (46.9%) cases and 10.3% of controls had a history of diarrhea in the last two weeks. Only 27.6% of cases and 21% of controls mothers had more than or equal to two pregnancies (Table 2).

### 3.3. Housing, Environmental, and Related Characteristics

The majority of cases (93.8%) and controls (81.4%) were living in houses with earthen floors and 93.8% of cases and 83.1% of controls were living in houses with walls made up of wood and mud. About 73.1% of cases and 93.4% of control's house had a window. More than 75% of cases and controls used charcoal and wood for cooking. Only 30.3% of cases and 3.8% of controls were carried on the back during cooking. About 94.5% cases and 63.8% had heard/been trained how to handle domestic smoking by health extension workers (Table 3).

### 3.4. Determinants of Under-Five Pneumonia

After adjusted for sociodemographic, child, and maternal characteristics of the children, multivariable logistic regression analysis identified child history of diarrhea during the last 15 days, mothers trained/heard about how to handle domestic smoking by health extension workers, and mother's compliance behavior on proper handwashing as factors significantly associated with under-five pneumonia (Table 4).

Accordingly, the odds of pneumonia was 6 (AOR = 6.183, 95% CI: 3.482, 10.977) times higher among under-five children who had a history of diarrhea during the past fifteen days when compared to their counterparts. In addition to that, the odds of pneumonia was also 5.8 (AOR = 5.814, 95% CI: 2.757, 12.261) times higher among children whose mother's did not hear/ were not trained about how to handle domestic smoking by health extension workers. The last but not the least variable which had a significant association with under-five pneumonia was handwashing practice. The odds of pneumonia among children whose mothers did not follow proper handwashing practice were 3.4 (AOR = 3.469, 95% CI: 1.753, 6.863) times higher than their counterparts.

## 4. Discussion

This study revealed that children who had a history of diarrhea during the last 15 days prior to data collection, improper handwashing practice by mothers/caregivers, and children whose mothers were not trained how to handle domestic smoking were determinants of under-five pneumonia at Gondar University Hospital.

A child who had a history of diarrhea during the last 2 weeks was 6 times more likely to get pneumonia as compared to a child who does not have a history of diarrhea. The result was similar to a study conducted in Ethiopia and Brazil [16, 31]. A study conducted in an urban area of Amhara region stated that children having a history of diarrhea (AOR = 3.06, 95% CI: 1.54, 6.11) were more likely to have pneumonia than their counterparts [16]. The reason might be due to the fact that previous episodes of diarrhea put the child at higher risk of contracting pneumonia through compromising their immunity. On the contrary, diarrhea is the determinant of pneumonia and vice versa as indicated in lancet series [32]. Therefore, to reduce the occurrence of an impact of these communicable diseases, integrated approaches can help reduce the burden and effects related to pneumonia and diarrhea.

Similarly, mothers/caregivers who did not apply proper handwashing practice were at higher odds of contracting pneumonia as compared to those who practiced proper handwashing. Studies conducted elsewhere revealed that proper handwashing with soap reduces the burden of pneumonia by 50% [33] and 16% of respiratory infections [34–36]. A meta-analysis conducted to determine the effect of enhanced hand hygiene on the morbidity of ventilator-associated pneumonia (VAP) revealed a pooled odds ratio of 2.23 (95% CI: 1.62, 3.07) [37]. The possible explanation might be improper handwashing predisposes to many diseases because many pathogenic bacteria are carried out by unclean hands. Promotion of proper handwashing has enormous benefits in terms of reducing the incidence and prevalence of infection like gastrointestinal and acute respiratory infections.

Moreover, children of mothers who did not hear or were not trained about how to handle domestic smoking by health extension workers were at higher risk of contracting pneumonia as compared to their counterpart. Domestic smoking results in indoor air pollution by emitting air pollutants from biomass fuels. As explained by different investigators, indoor air pollution caused by smoking cigarette and the use of biomass fuel increased the risk of contracting pneumonia [12, 29, 38]. For instance, Uddin et al. explained that children from indoor air polluted houses in Bangladesh had 5 times higher risk of developing pneumonia than children from relatively clean indoor air (AOR = 5.04; 95% CI: 2.41, 10.53) [29]. Similarly, a child whose parents used charcoal as main fuel was more likely to have pneumonia than those who did not use (AOR = 7.41; 95% CI: 2.75, 19.95) as presented by Fekadu et al. [12] and study from elsewhere (AOR = 2.09; 95% CI: 1.39–3.14) [38]. These pollutants adversely affect the respiratory tracts of under-five children [24, 29]. As a result, a mother who had been taught about how to handle these domestic smoking had a lesser risk of their children developing pneumonia. The possible explanation for this might be, nowadays, health extension workers found in the rural areas teach the community about the disadvantages of domestic smoking.

## 5. Limitation of the Study

This study is limited in terms of generalizability since the study was conducted in an exclusive hospital setting. Smaller sample size in certain categories reduced the precision of the study. Therefore, the use of this finding for any concern should account for the inherent limitation of the study.

## 6. Conclusion

In this study, child history of diarrhea within the last 15 days prior to data collection, mothers trained/heard about how to handle domestic smoking, and mothers' practice of handwashing were important factors associated with pneumonia among under-fives. We recommend dedicated, coordinated, and integrated actions for the prevention and control of diarrheal diseases. Moreover, enhancement of compliance with proper handwashing with soap among mothers and caregivers should be emphasized in addition to the appropriate use of fuel for domestic purposes.

## Figures and Tables

**Table 1:** Sociodemographic characteristics of under-five children, Gondar University Specialized Hospital, Northwest Ethiopia (*N* = 145 cases and 290 controls).

Variables	Cases (*n* = 145) (%)	Controls (*n* = 290) (%)
Age of child in months		
<12 months	61 (42.1)	166 (57.2)
≥12 months	84 (57.9)	124 (42.8)
Sex of child		
Male	71 (49.0)	151 (52.1)
Female	74 (51.0)	139 (47.9)
Residence		
Urban	91 (62.8)	240 (82.8)
Rural	54 (37.2)	50 (17.2)
Age of the mother		
15–24	89 (61.6)	181 (62.4)
25–34	29 (20.0)	76 (26.2)
≥35	27 (18.6)	33 (11.3)
Household monthly income		
<3687	88 (60.7)	149 (51.7)
≥3687	57 (39.3)	141 (48.3)
Mother's occupation		
Housewife	42 (29.0)	156 (53.8)
Government employee	40 (27.6)	84 (29.0)
Merchant	14 (9.7)	28 (9.7)
Others (farmer and student)	49 (33.8)	22 (7.6)
Father's occupation		
Government employee	47 (32.4)	98 (33.8)
Merchant	26 (17.9)	103 (35.5)
Farmer	56 (38.6)	62 (21.4)
Others (driver, student, private worker, and factory worker)	16 (11.1)	27 (9.3)
Family size		
<4	89 (61.4)	207 (71.4)
≥4	56 (38.6)	83 (28.6)

**Table 2:** children- and parental-related characteristics of respondents in Gondar University Specialized Hospital, Northwest Ethiopia, 2015 (*N* = 145 cases and *N* = 290 controls).

Variables	Cases (*n* = 145) (%)	Controls (*n* = 290) (%)
Exclusive breastfeeding history		
Yes	126 (86.9)	283 (97.6)
No	20 (13.1)	7 (2.4)
Pentavalent vaccine		
Not vaccinated	9 (6.2)	6 (2.9)
Fully vaccinated	136 (93.8)	284 (97.1)
Measles vaccine		
No	38 (26.2)	46 (15.9)
Yes	107 (73.8)	244 (84.1)
Illness of pneumonia within 2 weeks		
No	72 (49.7)	285 (98.3)
Yes	73 (50.3)	5 (1.7)
Illness of measles within 2 weeks		
No	137 (59.1)	287 (99.0)
Yes	8 (40.9)	3 (1.0)
Illness of URTIs within 2 weeks		
No	84 (57.1)	228 (78.6)
Yes	61 (42.1)	62 (21.4)
Child HIV status		
Negative	141 (97.2)	270 (93.1)
Positive	4 (2.8)	20 (6.9)
Child having repeated attack of pneumonia		
No	116 (80.0)	237 (81.7)
Yes	29 (20.0)	53 (18.3)

**Table 3:** Housing- and environmental-related characteristics of respondents in Gondar University Specialized Hospital Northwest Ethiopia, 2015 (*N* = 145 cases and *N* = 290 controls).

Variables	Cases (*n* = 145) (%)	Controls (*n* = 290) (%)
Place of cooking		
In kitchen	102 (70.3)	271 (93.45)
In house	43 (29.7)	19 (6.55)
Fuel used for cooking		
Charcoal/wood	115 (79.3)	223 (76.9)
Gas	—	6 (2.1)
Electricity	30 (20.7)	61 (21.0)
Proper handwashing practice		
No	44 (30.34)	24 (8.27)
Yes	101 (69.66)	266 (91.73)
A family history of smoking cigarette		
No	141 (97.2)	284 (97.9)
Yes	4 (2.8)	6 (2.1)

**Table 4:** Bivariable and multivariable logistic regression model for factors associated with under-five pneumonia, Gondar University Specialized Hospital, Northwest Ethiopia.

Variables	Pneumonia		COR, _95% CI_	AOR, _95% CI_
	Cases	Controls		
Place 0				
Urban	91	240	1	
Rural	54	50	2.848 (1.809–4.485)	—
Family size				
<4	89	207	1	
≥4	56	83	1.569 (1.031–2.389)	—
Age of child				
<12 months	61	166	1	
≥12 months	84	124	1.843 (1.231–2.760)	—
Measles vaccine				
Yes	107	244	1	
No	38	46	1.884 (1.159–3.063)	—
Exclusive breastfeeding				
Yes	126	283	1	
No	19	7	6.097 (2.500–14.925)	—
Diarrhea within the last 2 weeks				
No	77	260	1	1
Yes	68	30	7.654 (4.646–12.609)	6.183 (3.482, 10.977)^∗^
Measles within 2 weeks				
No	137	287	1	
Yes	8	3	5.586 (1.459–21.386)	—
Type of floor				
Cement	9	55	1	
Earth	136	235	3.573 (1.695–7.381)	—
Type of wall				
Cement	9	49	1	
Earth	136	241	3.072 (1.464–6.447)	—
Presence of window				
No	39	19	5.248 (2.902–9.491)	
Yes	106	271	1	—
Heard/trained how to handle domestic smoking				
No	18	74	2.417 (1.381–4.231)	5.814 (2.757, 12.261)^*∗*^
Yes	127	216	1	1
Handwashing practice				
No	44	24	4.828 (2.792–8.349)	3.469 (1.753, 6.863)^*∗*^
Yes	101	266	1	1

*Note.* ^*∗*^Significant at *p* value < 0.05.

## Data Availability

All data generated or analysed during this study are included in this published article.

## Abbreviations

AOR: Adjusted odds ratio
95% CI: 95% confidence interval
SPSS: Statistical package for social sciences
COR: Crude odds ratio.
